# Supplementary material for: Limits to the accurate and generalizable use of soundscapes to monitor biodiversity
Source: Nat Ecol Evol. 2023 Jul 31;7(9):1373–8. doi: 10.1038/s41559-023-02148-z (PMC10482675; doi:10.1038/s41559-023-02148-z)
Supplement: Supplementary file 1 — Supplementary Table 1. [file 41559_2023_2148_MOESM1_ESM.pdf]

nature ecology & evolution

Brief Communication

https://doi.org/10.1038/s41559-023-02148-z

Limits to the accurate and generalizable use of soundscapes to monitor biodiversity

In the format provided by the authors and unedited

Supplementary Information

Supplementary Table 1: List of all 60 scikit-maad v1.3 features used and the functions used to generate them – referred to as soundscape indices (SSIs) throughout the study. In all cases, default parameters were used, details of which can be found within the official documentation https://scikit-maad.github.io/.

Feature	scikit-maad v1.3 function	Feature	scikit-maad v1.3 function
ZCR	all_temporal_alpha_indices	EPS_KURT	all_spectral_alpha_indices
MEANt	all_temporal_alpha_indices	EPS_SKEW	all_spectral_alpha_indices
VARt	all_temporal_alpha_indices	ACI	all_spectral_alpha_indices
SKEWt	all_temporal_alpha_indices	NDSI	all_spectral_alpha_indices
KURTt	all_temporal_alpha_indices	rBA	all_spectral_alpha_indices
LEQt	all_temporal_alpha_indices	AnthroEnergy	all_spectral_alpha_indices
BGNt	all_temporal_alpha_indices	BioEnergy	all_spectral_alpha_indices
SNRt	all_temporal_alpha_indices	BI	all_spectral_alpha_indices
MED	all_temporal_alpha_indices	ROU	all_spectral_alpha_indices
Ht	all_temporal_alpha_indices	ADI	all_spectral_alpha_indices
ACTtFraction	all_temporal_alpha_indices	AEI	all_spectral_alpha_indices
ACTtCount	all_temporal_alpha_indices	LFC	all_spectral_alpha_indices
ACTtMean	all_temporal_alpha_indices	MFC	all_spectral_alpha_indices
EVNtFraction	all_temporal_alpha_indices	HFC	all_spectral_alpha_indices
EVNtMean	all_temporal_alpha_indices	ACTspFract	all_spectral_alpha_indices
EVNtCount	all_temporal_alpha_indices	ACTspCount	all_spectral_alpha_indices
MEANf	all_spectral_alpha_indices	ACTspMean	all_spectral_alpha_indices
VARf	all_spectral_alpha_indices	EVNspFract	all_spectral_alpha_indices
SKEWf	all_spectral_alpha_indices	EVNspMean	all_spectral_alpha_indices
KURTf	all_spectral_alpha_indices	EVNspCount	all_spectral_alpha_indices
NBPEAKS	all_spectral_alpha_indices	TFSD	all_spectral_alpha_indices
LEQf	all_spectral_alpha_indices	H_Havrda	all_spectral_alpha_indices
ENRf	all_spectral_alpha_indices	H_Renyi	all_spectral_alpha_indices
BGNf	all_spectral_alpha_indices	H_pairedShannon	all_spectral_alpha_indices
SNRf	all_spectral_alpha_indices	H_gamma	all_spectral_alpha_indices
Hf	all_spectral_alpha_indices	H_GiniSimpson	all_spectral_alpha_indices
EAS	all_spectral_alpha_indices	RAOQ	all_spectral_alpha_indices
ECU	all_spectral_alpha_indices	AGI	all_spectral_alpha_indices
ECV	all_spectral_alpha_indices	ROItotal	all_spectral_alpha_indices
EPS	all_spectral_alpha_indices	ROIcover	all_spectral_alpha_indices
