# Supplementary material for: Limits to the accurate and generalizable use of soundscapes to monitor biodiversity
Source: Nat Ecol Evol. 2023 Jul 31;7(9):1373–8. doi: 10.1038/s41559-023-02148-z (PMC10482675; doi:10.1038/s41559-023-02148-z)
Supplement: Supplementary file 2 — Reporting Summary [file 41559_2023_2148_MOESM2_ESM.pdf]

nature portfolio

Corresponding author(s): Sarab S Sethi

Last updated by author(s): 26/06/2023

Reporting Summary

Nature Portfolio wishes to improve the reproducibility of the work that we publish. This form provides structure for consistency and transparency in reporting. For further information on Nature Portfolio policies, see our Editorial Policies and the Editorial Policy Checklist.

Statistics

For all statistical analyses, confirm that the following items are present in the figure legend, table legend, main text, or Methods section.

n/a	Confirmed	
☐	☒	The exact sample size (*n*) for each experimental group/condition, given as a discrete number and unit of measurement
☐	☒	A statement on whether measurements were taken from distinct samples or whether the same sample was measured repeatedly
☐	☒	The statistical test(s) used AND whether they are one- or two-sided<br>*Only common tests should be described solely by name; describe more complex techniques in the Methods section.*
☒	☐	A description of all covariates tested
☐	☒	A description of any assumptions or corrections, such as tests of normality and adjustment for multiple comparisons
☐	☒	A full description of the statistical parameters including central tendency (e.g. means) or other basic estimates (e.g. regression coefficient) AND variation (e.g. standard deviation) or associated estimates of uncertainty (e.g. confidence intervals)
☐	☒	For null hypothesis testing, the test statistic (e.g. *F*, *t*, *r*) with confidence intervals, effect sizes, degrees of freedom and *P* value noted<br>*Give P values as exact values whenever suitable.*
☒	☐	For Bayesian analysis, information on the choice of priors and Markov chain Monte Carlo settings
☒	☐	For hierarchical and complex designs, identification of the appropriate level for tests and full reporting of outcomes
☐	☒	Estimates of effect sizes (e.g. Cohen's *d*, Pearson's *r*), indicating how they were calculated

Our web collection on statistics for biologists contains articles on many of the points above.

Software and code

Policy information about availability of computer code

Data collection	No software was used for data collection
Data analysis	Software for analysis can be found at https://github.com/sarabsethi/sscape-avian-div-generalisability (versioned at https://zenodo.org/record/7845899)

For manuscripts utilizing custom algorithms or software that are central to the research but not yet described in published literature, software must be made available to editors and reviewers. We strongly encourage code deposition in a community repository (e.g. GitHub). See the Nature Portfolio guidelines for submitting code & software for further information.

Data

Policy information about availability of data

All manuscripts must include a data availability statement. This statement should provide the following information, where applicable:

- Accession codes, unique identifiers, or web links for publicly available datasets
- A description of any restrictions on data availability
- For clinical datasets or third party data, please ensure that the statement adheres to our policy

Processed acoustic features and avian community data for all point counts is published at https://doi.org/10.5281/zenodo.7410357. Code with instructions to reproduce analyses, results, and figures can be found at https://zenodo.org/record/7845899.

Human research participants

Policy information about studies involving human research participants and Sex and Gender in Research.

Reporting on sex and gender	N/A
Population characteristics	N/A
Recruitment	N/A
Ethics oversight	N/A

Note that full information on the approval of the study protocol must also be provided in the manuscript.

Field-specific reporting

Please select the one below that is the best fit for your research. If you are not sure, read the appropriate sections before making your selection.

☒ Life sciences ☐ Behavioural & social sciences ☐ Ecological, evolutionary & environmental sciences

For a reference copy of the document with all sections, see nature.com/documents/nr-reporting-summary-flat.pdf

Life sciences study design

All studies must disclose on these points even when the disclosure is negative.

Sample size

India: Data was collected from 91 sites in the Western Ghats between March 2020 and May 2021. Sites were a mixture of montane wet evergreen and semi-evergreen forests (29), montane grasslands (8), moist deciduous forests (7), timber plantations (17), tea plantations (20), agricultural land (8), and settlements (2).
147 15-minute point counts were conducted between 6-10am at each site (mean 1.6 per site). A variable-distance point count approach was followed, and all bird species heard, seen, and those that flew over (primarily raptor species) were noted. All point counts were carried out between 6am and 10am (timing of high avian activity) at each location.
Single channel audio was recorded during each point count using an Audiomoth device6. Recordings were saved in WAV format at a sampling rate of 48 kHz.
Malaysia: Data was collected from a varied tropical landscape at the Stability of Altered Forest Ecosystems (SAFE) project24 in Sabah between March 2018 and February 2020. The 14 sites spanned a degradation gradient: two in protected old growth forest, two in a protected riparian reserve, six in selectively logged forest (logging events in 1970s and early 2000s), two in salvage logged forest (last logged in early 2010s), and two in oil palm plantations. Sites had a mean separation distance of 7.6km, with the closest two being 583m apart.
977 20-minute avifaunal point counts were performed across 24 hours of the day at each site (59-80 per site). During point counts all visual or aural encounters of avifaunal species within a 10m radius of the sampling site were recorded. Species identifications and names were validated using the Global Biodiversity Information Facility25 (GBIF). A total of 216 avifaunal species were recorded across all point counts.
Single channel audio was recorded during each point count with a Tascam DR-05 recorder mounted to a tripod at chest height (nominal input level -20 dBV, range 20Hz-22kHz). Recordings were saved in WAV format at a sampling rate of 44.1k kHz.
Taiwan: Data was collected from 16 tea plantations in the Alishan tea district, located in Chiayi County of Taiwan, between January and November 2022. The tea plantations spanned an elevation gradient from 816-1464 m and were surrounded by secondary broadleaf forests or coniferous plantations.
Stereo audio was recorded at a sampling rate of 44.1kHz for one of every fifteen minutes at each site throughout the sampling period. Wildlife Acoustics Song Meter 4 devices were used (mounted to a tree truck or tripod at chest height) and data was saved in WAV format.
176 10-minute avifaunal point counts were conducted in total, on average once per site per month. Every survey was conducted within 3 hours after sunrise. During point counts, the species of every bird individual visually or aurally detected was recorded and its horizontal distance from the observer was estimated as either 0-25m, 25-50m, 50-100m, >100m or flying over. Our process for matching audio recordings to point counts is provided later in the Methods.
USA: Data was collected from a single site in a temperate forest at Sapsucker Woods, Ithaca from January 2016 to December 2021.
Audio was recorded continuously using a Gras-41AC precision microphone mounted at chest height and digitised with a Barix Instreamer ADC at a sampling rate of 48 kHz. Files were saved in 15-minute chunks and in WAV format.
In the absence of standardised point counts across such a long duration, we used eBird checklists to determine avian communities (only possible since Sapsucker Woods is a major hotspot for eBird data). Data was filtered to only keep checklists which were complete, of the “travelling” or “stationary” types, located within 200m of the recording location, and shorter than 30 minutes. We combined occurrence data from all eBird checklists that started during each audio recording to create one pseudo point count per audio file. Since we only use occurrence data, double counting of the same species was not an issue. This resulted in a total of 6,734 point counts.

Data exclusions	N/A
Replication	N/A
Randomization	N/A

Blinding | N/A

Reporting for specific materials, systems and methods

We require information from authors about some types of materials, experimental systems and methods used in many studies. Here, indicate whether each material, system or method listed is relevant to your study. If you are not sure if a list item applies to your research, read the appropriate section before selecting a response.

Materials & experimental systems

n/a	Involved in the study
☒	☐ Antibodies
☒	☐ Eukaryotic cell lines
☒	☐ Palaeontology and archaeology
☒	☐ Animals and other organisms
☒	☐ Clinical data
☒	☐ Dual use research of concern

Methods

n/a	Involved in the study
☒	☐ ChIP-seq
☒	☐ Flow cytometry
☒	☐ MRI-based neuroimaging
